# Rastreamento do câncer de mama: modelo de melhoria do acesso pelo uso de mamógrafos móveis

**DOI:** 10.26633/RPSP.2019.19

**Published:** 2019-02-06

**Authors:** Gerson Nunes da Cunha, Cid Manso de Mello Vianna, Gabriela Bittencourt Gonzalez Mosegui, Marcus Paulo Rodrigues da Silva, Fernando Nagib Jardim

**Affiliations:** 1 Faculdade de Tecnologia do Estado do Rio de Janeiro Faculdade de Tecnologia do Estado do Rio de Janeiro PetrópolisRJ Brasil Faculdade de Tecnologia do Estado do Rio de Janeiro, Petrópolis (RJ), Brasil.; 2 Instituto de Medicina Social Instituto de Medicina Social Universidade do Estado do Rio de Janeiro (UERJ) Rio de JaneiroRJ Brasil Universidade do Estado do Rio de Janeiro (UERJ), Instituto de Medicina Social, Rio de Janeiro (RJ), Brasil.; 3 Instituto de Saúde Coletiva Instituto de Saúde Coletiva Universidade Federal Fluminense (UFF) Rio de JaneiroRJ Brasil Universidade Federal Fluminense (UFF), Instituto de Saúde Coletiva, Rio de Janeiro (RJ), Brasil.

**Keywords:** Mamografia, neoplasias da mama, política pública, logística, Mammography, breast neoplasms, public policy, logistics, Mamografía, neoplasias de la mama, política pública, logística

## Abstract

**Objetivo.:**

Investigar o impacto do uso combinado de mamógrafos fixos e móveis para racionalizar a gestão dos programas de rastreamento do câncer de mama, a fim de ampliar a cobertura à população.

**Métodos.:**

Realizou-se um estudo observacional utilizando um modelo computacional baseado em agentes. O modelo foi utilizado para simular a cobertura por rastreamento do câncer de mama na região serrana do Rio de Janeiro, Brasil, onde existem 22 mamógrafos fixos instalados. Foram estimados o número e a distribuição de mamógrafos fixos e móveis e o número de exames por dia necessário para alcançar uma cobertura de 100% e uma cobertura de 60% da população da região no biênio 2015-2016.

**Resultados.:**

Para o período de 2 anos, determinou-se que a cobertura de 60% da população seria alcançada com oito mamógrafos, cinco fixos e três móveis. Para um cenário onde 100% da população elegível faria o exame, haveria necessidade de sete mamógrafos fixos e quatro mamógrafos móveis, totalizando 11 equipamentos na região serrana. A cobertura real de mamografia na região para o biênio 2015-2016 foi de 36,4%, com 22 mamógrafos realizando quatro exames por dia.

**Conclusões.:**

A simulação mostrou que seria possível reduzir pela metade o número de equipamentos existentes na região estudada, garantindo 100% de cobertura. O uso de um maior número de mamógrafos móveis facilitaria o acesso da população nos municípios sem mamógrafos e em áreas rurais.

## Abstract (en)

**Objective.:**

To investigate the impact of combined use of fixed and mobile mammography units to rationalize the management of breast cancer screening programs and increase population coverage.

**Methods.:**

An observational study was performed using agents-based modeling. The model was used to simulate breast cancer screening coverage in a specific region in the state of Rio de Janeiro (*região serrana*) where 22 fixed mammography units are installed. The number and distribution of fixed and mobile units, as well as the required number of daily exams, were estimated considering a population coverage of 100% and 60% in the region for the 2015-2016 biennium.

**Results.:**

For the two-year period, a 60% population coverage could be reached with eight mammography units (five fixed and three mobile). Considering a scenario in which 100% of the eligible population would undergo screening, 11 units would be required (seven fixed and four mobile units). The actual coverage in the region for the 2015-2016 biennium was 36.4%, with 22 mammography units performing four exams daily.

**Conclusions.:**

The present simulation showed that it would be possible to reduce by half the number of mammography units in the region, ensuring 100% coverage. Adding more mobile units would facilitate access by the population from cities without installed mammography units and from rural areas.

## RESUMEN

**Objetivo.:**

Investigar la repercusión del uso conjunto de mamógrafos fijos y móviles para racionalizar la gestión de los programas de seguimiento del cáncer de mama, con el objeto de ampliar la cobertura de la población.

**Métodos.:**

Se realizó un estudio de observación con un modelo computacional basado en agentes. El modelo se utilizó para simular la cobertura mediante el seguimiento del cáncer de mama en la región serrana del estado de Rio de Janeiro (Brasil), donde se han instalado 22 mamógrafos fijos. Se estimaron el número y la distribución de mamógrafos fijos y móviles y el número de exámenes por día necesarios para lograr una cobertura de un 100% y de un 60% de la población de la región serrana en el bienio 2015-2016.

**Resultados.:**

Se determinó que, en el bienio objeto de estudio, se alcanzaría una cobertura de un 60% de la población con ocho mamógrafos (cinco fijos y tres móviles). En un escenario en el cual se haría el examen a un 100% de la población seleccionada, se necesitaría un total de 11 mamógrafos (siete fijos y cuatro móviles) en la región serrana. En el bienio citado, la cobertura real de la mamografía en dicha región fue de 36,4%, con la realización de cuatro exámenes diarios con 22 mamógrafos.

**Conclusiones.:**

Esta simulación mostró que sería posible reducir a la mitad el número de equipos existentes en la región estudiada y garantizar un 100% de cobertura. El uso de un mayor número de mamógrafos móviles facilitaría el acceso de la población en los municipios carentes de ese equipo y en las zonas rurales.

O câncer de mama é o tumor mais comum entre mulheres em todo o mundo (1, 2). A detecção precoce da doença, ainda nos seus estágios iniciais, aumenta a chance de sucesso no tratamento, além de permitir a utilização de uma ampla variedade de intervenções terapêuticas (1, 3, 4). Nesse contexto, a atenção primária à saúde (APS) assume uma importância estratégica nas ações de prevenção e detecção precoce do controle do câncer de mama.

A dificuldade de acesso ao exame de mamografia faz com que muitas mulheres só consigam o diagnóstico da doença em estado avançado. Com isso, elas são severamente punidas com tratamentos mais caros, mais invasivos e, na maioria das vezes, menos eficientes. Os estados mais avançados estão associados a menor sobrevida e aumento da chance de mastectomia (3-5).

No Brasil, a política brasileira de rastreamento de câncer está baseada na pactuação entre Colegiados de Gestão Regionais (6). Conforme essa pactuação, as mulheres residentes em municípios sem mamógrafos, ou que não possuem equipamentos suficientes para examinar toda a população elegível, devem ser atendidas em outras cidades. A gestão dessa pactuação nem sempre tem apresentado resultados eficientes, ou seja – muitas mulheres permanecem sem atendimento, não necessariamente pela falta de equipamentos, mas pela ausência de especialistas para solicitação dos exames, pelo número baixo de exames disponibilizados ou mesmo por dificuldade de acesso, especialmente no caso daquelas que residem na zona rural (6-8).

Na tentativa de diminuir essas desigualdades, principalmente em regiões onde a aquisição de equipamentos fixos não é viável, a utilização de mamógrafos móveis surge como alternativa para aumentar a realização de exames de rastreamento do câncer de mama (6, 9-11). O objetivo deste trabalho foi investigar o impacto do uso combinado de mamógrafos fixos e móveis para racionalizar a gestão dos programas de rastreamento do câncer de mama, a fim de ampliar a cobertura à população.

## MATERIAIS E MÉTODOS

O presente estudo observacional foi realizado em duas etapas. Na primeira, foi feito um levantamento da situação atual do rastreamento de câncer de mama no estado do Rio de Janeiro a partir da coleta de informações disponíveis em diversos bancos de dados oficiais do governo. As informações sobre população-alvo foram obtidas do censo de 2010 do Instituto Brasileiro de Geografia e Estatística (IBGE) (12). O número de mamógrafos existentes foi obtido a partir do Cadastro Nacional de Estabelecimentos de Saúde (CNES) (13).

A região serrana do estado foi escolhida como modelo para a segunda etapa do estudo em função do perfil de distribuição dos mamógrafos fixos, da alta capacidade ociosa e da baixa cobertura alcançada. Dos 16 municípios dessa região, conforme mostra a tabela 1, oito concentram todos os mamógrafos fixos, tornando os demais municípios dependentes da pactuação do Colegiado Regional, promovendo um modelo de gestão ineficiente. A população urbana e rural estimada nessa região para o biênio 2015-2016 foi projetada utilizando os parâmetros de crescimento populacional disponibilizados pelo IBGE (12, 13).

A capacidade máxima de exames por mamógrafo fixo foi calculada conforme nota técnica do Instituto Nacional do Câncer (14): produção anual = 3 exames/hora * turno trabalho de 8h * 22 dias * 12 meses * desempenho de 80% = 5 069 mamografias/ano. Os dados da produção mamográfica dos municípios da região serrana foram retirados do CNES e dos bancos de dados do Sistema Único de Saúde (SUS) para o biênio 2015-2016 (15).

Para aperfeiçoar a cobertura do rastreamento por mamografia, criou-se um modelo computacional que simulou diversos cenários hipotéticos do uso de mamógrafos fixos e móveis. Simulou-se o número de exames por dia para cada mamógrafo, calculando a cobertura da mamografia na região de acordo com a população elegível e as características dos municípios na região serrana. Para o desenvolvimento desse modelo, foi utilizada a modelagem baseada em agentes (*agent-based modeling*, ABM). Essa é uma abordagem essencialmente descentralizada e focada no comportamento dos elementos individualizados do sistema (16).

Os agentes compreendem as mulheres da população alvo, os mamógrafos (fixos e móveis), os municípios e as estradas (rotas) entre esses municípios. Eles atuam no sistema de acordo com um comportamento próprio, inseridos nos seus ambientes. O comportamento global é resultado das interações entre todos os agentes individuais e dos inter-relacionamentos existentes.

Uma das principais características desses modelos é serem adaptáveis às necessidades do problema, com parâmetros que podem ser configurados em tempo de execução, sem a necessidade de modificações na estrutura implementada. Dessa forma, é possível simular cenários distintos modificando apenas os valores das variáveis dinâmicas do modelo.

A modelagem possui uma interface principal, onde são definidos os parâmetros de configuração e execução das simulações. As relações entre todos os agentes que são afetados pelos parâmetros dinâmicos são configuradas nessa interface. Esses parâmetros partem do pressuposto de que, como os equipamentos fixos estão localizados na zona urbana das cidades, o deslocamento das usuárias pode ser uma das causas para a baixa realização de exames (17, 18).

O modelo considera a região onde a usuária reside (urbana ou rural) como uma variável importante na modelagem. Durante a execução do modelo, os mamógrafos móveis foram alocados tanto em áreas urbanas quanto rurais, visando a melhorar a equidade de acesso (19, 20). Para cada mamógrafo simulado foram coletadas informações sobre número de exames realizados e período de tempo pelo qual os equipamentos poderiam ficar inativos devido a interrupções não programadas e manutenções preventivas.

A movimentação dos mamógrafos móveis foi modelada ao longo das estradas que representam a topologia de ligação entre os municípios. Outro parâmetro importante foi o tempo de permanência dos mamógrafos móveis nas cidades. A escolha desse tempo influencia o número de deslocamentos, os custos e a equidade de acesso. A fim de tornar mais equânime e “justa” a visita aos municípios, foi implementado o mecanismo de rodízio, garantindo a visita a todos os municípios antes do retorno a um município anteriormente visitado.

A otimização do modelo depende do critério a ser empregado para decidir as rotas de cada mamógrafo móvel, devido à sua influência direta na extensão de cobertura a ser alcançada. Os critérios testados na escolha do próximo município a ser visitado foram: percentual da população ainda não rastreada, maior número absoluto de mulheres sem rastreamento; cidades com menor população, diminuindo o número de retornos; e menor distância entre as cidades. Para este último foi implementada a versão clássica do algoritmo de Dijkstra para cálculo da menor distância entre os municípios (21), além de uma variação desse algoritmo, associando ao custo da aresta uma função baseada na distância e na população a ser rastreada nos munícipios.

Todas as entradas da solução foram parametrizadas, possibilitando a simulação de diferentes cenários e fornecendo informações importantes para a tomada de decisões. Dentre os diversos cenários possíveis, foram executadas simulações na tentativa de encontrar soluções para questões específicas, como número necessário de equipamentos, tempo de permanência e melhor forma de deslocamento entre as cidades. O percentual pactuado de mamógrafos fixos foi definido e alterado durante a execução das simulações, possibilitando encontrar o número de mamógrafos móveis necessário para o cenário escolhido. A influência isolada e combinada de alguns parâmetros também foi estudada na análise de sensibilidade.

## RESULTADOS

Dos 22 equipamentos instalados na região serrana do estado do Rio de Janeiro, 15 estão concentrados em apenas três cidades: Nova Friburgo (oito), Petrópolis (quatro) e Cachoeiras de Macacu (três). Os outros sete mamógrafos estão localizados em cinco cidades. Oito cidades não dispõem de equipamento. No biênio 2015-2016, apenas 36,4% da população elegível na região serrana realizou o exame de mamografia.

**FIGURA 1:**
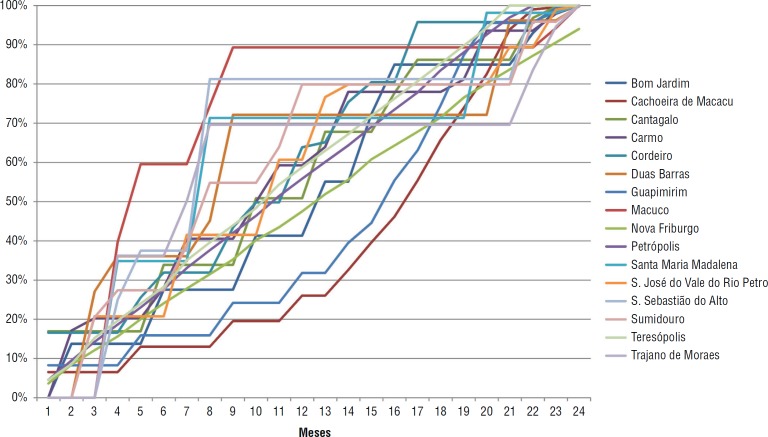
Evolução hipotética da cobertura do rastreamento do câncer de mama na região serrana do estado do Rio de Janeiro ao longo de 2 anos após redistribuição de mamógrafos fixos e móveis

A tabela 1 apresenta a situação atual do rastreamento na região serrana do Estado do Rio de Janeiro. Ela inclui o tamanho estimado da população elegível, o número de mamógrafos existentes, o número de exames e a cobertura possíveis, o número de exames realizados no biênio 2015-2016 e a respectiva cobertura alcançada em cada município da região serrana. Utilizou-se a produção real de cada mamógrafo fixo (13) e a população elegível (12) para o cálculo da cobertura alcançada. A cobertura possível foi calculada considerando-se o número de mamógrafos fixos existentes (13), a produção estabelecida por norma técnica do INCA (14) e a população elegível (12). Como os exames são totalizados na cidade onde foram realizados, e não no domicílio de origem das pacientes, os municípios sem equipamentos fixos têm a quantidade de exames zerada.

No biênio 2015-2016, realizaram-se 39 315 exames, correspondendo a 36,4% da população elegível e apenas 19,9% da capacidade total. Com esses valores, a produção diária foi de 4,8 exames, e não de 24, como recomendado pela Nota Técnica do INCA (14). Esses dados mostram que o sistema de rastreamento do Colegiado Regional não atingiu os resultados pactuados, mesmo contando com número de equipamentos bem superior ao que realmente seria necessário.

As simulações realizadas mostraram que seriam necessários apenas 11 mamógrafos, sendo sete fixos e quatro móveis, para alcançar 100% da população elegível. Esse número é a metade do que existe atualmente.

Considerando o cenário de pactuação de 60% de cobertura, como proposto no Pacto pela Vida (22), seriam necessários apenas oito mamógrafos: cinco fixos instalados (dois na cidade de Nova Friburgo, dois na cidade de Petrópolis e um em Teresópolis) e mais três móveis. Esse número é 64% menor do que o número de mamógrafos existentes. Na figura 1 é possível observar que a cobertura a 100% da população em todos os municípios poderia ser alcançada no final do ciclo de 2 anos, considerando a nova distribuição de mamógrafos fixos e móveis.

### Análise de sensibilidade

A distribuição alcançada pela simulação foi de sete mamógrafos fixos e quatro móveis considerando 24 exames por dia, conforme preconizado pela Nota Técnica do INCA (14). Esse número de exames pressupõe o uso de 80% da capacidade dos mamógrafos. Para validar a configuração alcançada, testou-se um cenário de cobertura de no mínimo 60% da população do Colegiado, como estabelecido no Pacto pela Vida (22, 23). Esse valor é aproximadamente duas vezes superior ao realizado no biênio 2015-2016 pelos 22 mamógrafos fixos existentes na região serrana.

**FIGURA 2:**
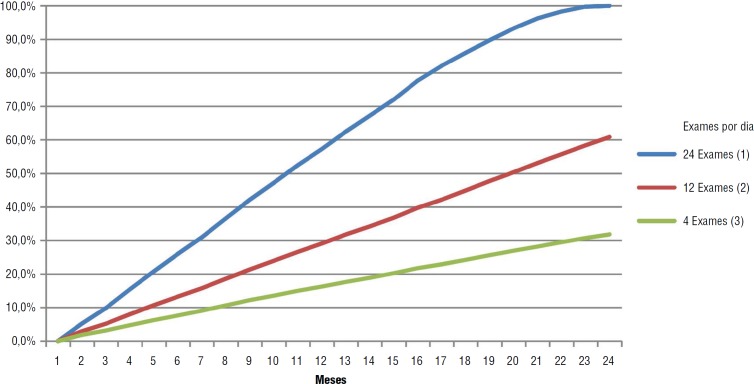
Cobertura total do rastreamento do câncer de mama em função da produção diária considerando as características da região serrana do estado do Rio de Janeiro^a^

^a^ 1) Produção com sete mamógrafos fixos e quatro móveis realizando 24 exames por dia; 2) produção com sete mamógrafos fixos e quatro móveis realizando 12 exames por dia; e 3) produção real do biênio 2015-2016 na região serrana com 22 mamógrafos instalados realizando quatro exames por dia.

A simulação mostrou que, para essa cobertura, seriam necessários apenas 50% dos exames recomendado pelo INCA, ou seja, 12 exames dia. Nesse contexto, a configuração proposta alcançaria as metas do Pacto pela Vida com apenas 40% da capacidade dos mamógrafos. A figura 2 apresenta os resultados encontrados nas três configurações de produção diária.

As taxas de cobertura alcançadas com quatro e 12 exames têm relação direta com o número de mamógrafos fixos existentes nas cidades mais populosas. A produção diária pode ser variada nas simulações, na tentativa de definir um valor mínimo para atender a demanda estabelecida com o número de equipamentos móveis disponíveis.

**TABELA 1:** Cobertura mamográfica real e modelada na região serrana do estado do Rio de Janeiro, Brasil, 2015-2016

Municípios	População elegível (n)	Mamógrafos fixos (n)	Exames possíveis (n)	Cobertura possível^a^ (%)	Exames realizados^b^ (n)	Cobertura alcançada^c^ (%)
Bom Jardim	2 675	0	0	0,0	0	0,0
Cachoeiras de Macacu	5 658	3	30 414	537,5	0	0,0
Cantagalo	2 174	0	0	0,0	441	20,3
Carmo	1 969	1	5 069	257,4	70	3,6
Cordeiro	2 405	1	10 138	421,5	4 535	188,6
Duas Barras	1 021	0	0	0,0	0	0,0
Guapimirim	4 822	2	20 276	420,5	0	0,0
Macuco	619	0	0	0,0	0	0,0
Nova Friburgo	23 604	8	60 828	257,7	8 856	37,5
Petrópolis	37 724	4	40 552	107,5	15 725	41,7
Santa Maria Madalena	1 145	0	0	0,0	0	0,0
São José do Vale do Rio Preto	1 923	1	10 138	527,2	1 532	79,7
São Sebastião do Alto	982	0	0	0,0	0	0,0
Sumidouro	1 345	0	0	0,0	0	0,0
Teresópolis	18 929	2	20 276	107,1	8 156	43,1
Trajano de Morais	1 103	0	0	0,0	0	0,0
Colegiado	108 098	22	197 691	182,9	39 315	36,4

***Fonte:*** Viana et al. (8) e Ministério da Saúde (9, 11).

a Cobertura possível em relação a número de mamógrafos fixos.

b Os exames realizados são computados na cidade onde foram realizados e não na cidade de origem, por isso os valores zerados em alguns municípios.

c Cobertura alcançada em relação a população elegível e número de exames realizados.

Nas análises de sensibilidade efetuadas, também foi possível considerar a realização de exames nos finais de semana e verificar o impacto nos resultados encontrados. Com a inclusão do sábado na agenda de realização de exames, tem-se um dia a mais disponível, o que representa um acréscimo de 20% na oferta de exames. A realização de exames nos finais de semana, por sua vez, merece um estudo mais detalhado. Isso porque, embora possa facilitar o acesso e aumentar o número de exames, essa ação exige contrapartidas administrativas, dentre elas a disponibilidade dos profissionais e a avaliação dos custos associados à sua operação.

## DISCUSSÃO

A prevenção precoce do câncer de mama envolve a necessidade de aumentar o acesso ao rastreamento, assim como a disponibilização de informações à população sobre os fatores de risco para o câncer e de estratégias para diminuir a exposição aos mesmos. Essa prevenção perpassa todos os níveis de atenção à saúde, mas é na APS que se torna possível um maior alcance das ações, em função de sua abordagem mais próxima da população, na ótica da promoção da saúde. No Brasil, a pactuação nos Colegiados Regionais de Saúde deveria ser a solução para atender a demanda de cada região, mas, pelo exposto, não se mostra por si só o instrumento definitivo para garantir a ­cobertura desejada do exame de rastreamento de câncer de mama.

A política atual de facilitar o rastreamento mamográfico com base na aquisição de mamógrafos fixos revela-se ineficiente. Conforme visto, ainda que o Colegiado da região serrana disponibilize 22 mamógrafos fixos, número suficiente para examinar quase o dobro da população existente, somente 36,4% da população elegível foi atendida no biênio 2015-2016. Essa baixa cobertura e ineficiência é produto de decisões colegiadas feitas sem critérios adequados sobre a real necessidade da região, não só em termos de equipamentos, mas também de médicos. Além disso, deveriam ser realizadas ações para facilitar o acesso e aumentar a adesão da população.

Para o estado do Rio de Janeiro, esses números não são muito diferentes. No biênio 2015-2016, apenas 24,8% da população elegível realizou a mamografia, apesar da capacidade instalada suficiente para o rastreamento de 86,8% da população elegível. Cinquenta munícipios concentram 159 mamógrafos e 42 não possuem nenhum, tornando-se totalmente dependentes da ­pactuação em suas regiões colegiadas. Apenas 12 municípios (13% do total de municípios) alcançaram 60% de cobertura da população, enquanto 69 municípios (75% do total de municípios) não conseguiram sequer atingir 30% de cobertura.

A opção pela aquisição de equipamentos móveis melhora a adesão aos programas de rastreamento, possibilitando que mulheres residentes em municípios de menor porte, ou em áreas rurais, possam ser atendidas em locais mais próximos a suas residências. O rastreamento ativo aumenta a equidade de acesso ao exame mamográfico dentro dos prazos estabelecidos pelo Ministério da Saúde e a realização de diagnósticos mais precoces (19). O governo do estado do Rio de Janeiro reconhece a deficiência da rede para detecção precoce do câncer de mama pela baixa produção (24).

O objeto deste trabalho foi estimar uma oferta mais eficiente de cobertura através da utilização de mamógrafos móveis, racionalizando o uso de recursos e facilitando o acesso da população-alvo. Deve-se observar, por sua vez, que, embora proponha uma distribuição mais eficiente dos mamógrafos fixos e móveis, o modelo não tem como garantir que as metas de rastreamento sejam atingidas. Existem outros fatores, que não foram objeto da simulação, que podem influenciar esses resultados.

A necessidade de deslocamento das usuárias entre cidades e entre áreas rurais e urbanas, a falta de equipamentos e recursos humanos e problemas culturais da população, como preconceito e falta de informação a respeito da doença, são fatores determinantes para o baixo número de exames realizados. A falta de médicos também é um agravante, pois há enorme carência de especialistas para solicitar e realizar os exames e, principalmente, para acompanhar as pacientes que tenham diagnóstico positivo (4). Ainda assim, a racionalização dos recursos ataca dois dos principais problemas da efetividade do rastreamento: o número de profissionais de saúde necessários; e a ausência de mamógrafos onde as mulheres vivem.

Análises como as do presente estudo, baseadas nos resultados de simulações, podem ser úteis para reforçar a necessidade de políticas de saúde que, além de disponibilizarem os exames, conscientizem a população das vantagens de aderir ao programa de rastreamento mamográfico. Sem essas ações, a taxa de efetividade de utilização dos mamógrafos tende a continuar em níveis bem abaixo dos estipulados no Pacto pela Vida (22, 23).

A maioria dos estudos realizados para analisar o papel de mamógrafos móveis no rastreamento de câncer procura demonstrar que o seu uso mais intenso promove uma maior cobertura e equidade (19, 25, 26). Tais estudos analisam aspectos organizacionais e procedimentos relacionados ao uso mais eficiente de cada um dos mamógrafos (27, 28). Nenhum deles avalia qual a distribuição mais eficiente para uma determinada região, como o exemplo aqui apresentado.

O modelo desenvolvido neste trabalho pode ser uma ferramenta de apoio à tomada de decisão, pois permite que diversos cenários sejam simulados e analisados. Os resultados alcançados pela simulação mostraram-se úteis para programas de rastreamento mamográfico, possibilitando ainda que a pactuação dentro dos Colegiados de Saúde seja mais eficiente. A reavaliação da forma de aquisição de mamógrafos deve ser a base do processo de reestruturação das políticas de rastreamento do câncer de mama no Brasil.

O estudo, por se tratar de uma simulação, propõe algumas simplificações que podem representar limitações. Considerou-se que todos os mamógrafos estariam em operação durante todo o horizonte temporal. Outra limitação relevante foi considerar que toda infraestrutura de recursos humanos também estaria disponível, sem que nenhum exame deixasse de ser realizado por falta de profissionais de saúde.

Outro aspecto limitante é a abrangência das áreas de cobertura. O atendimento móvel em áreas urbanas ou rurais extensas poderia produzir efeitos semelhantes à instalação de mamógrafos fixos, uma vez que a distância dos domicílios em relação aos pontos de atendimento dos mamógrafos móveis poderia continuar dificultando o acesso das usuárias.

Por sua vez, o modelo pode ser usado como ferramenta na proposição de novas políticas de aquisição de equipamentos. É possível realizar simulações e estimar o número de mamógrafos necessários, como foi feito para a região serrana, para minimizar o desperdício de recursos e reforçar a pactuação entre os municípios. Finalmente, vale ressaltar que o estudo pode ser facilmente adaptado a outros problemas de logística na área de saúde. Diversos programas utilizam unidades móveis para a prestação de serviços, tais como tomografia, ultrassonografia e atendimento odontológico. Como a modelagem foi baseada em agentes, seria necessário apenas identificar e modelar as características das novas entidades de cada problema.

## Conclusões

A simulação e análise de diferentes cenários demonstrou a ineficiência do modelo de gestão atual baseado na aquisição e instalação de mamógrafos fixos nas grandes cidades. Uma combinação mais racional do uso de mamógrafos fixos e móveis permitiu projetar uma redução pela metade do número de equipamentos existentes, garantindo 100% de oferta de cobertura à população de mulheres residentes na região serrana do estado do Rio de Janeiro. O uso de um número maior de mamógrafos móveis facilitaria o acesso da população nos municípios sem mamógrafos e em áreas rurais.

## Contribuição dos autores.

GNC e CMMV conceberam e desenharam a pesquisa. GNC, FNJ, GBGM e MPRS realizaram a obtenção de dados e redigiram o artigo. Todos os autores analisaram e interpretaram os dados, revisaram criticamente o conteúdo e revisaram e aprovaram a versão final.

## Conflitos de interesse.

Nada declarado pelos autores.
